# Management challenges in metachronous bilateral breast cancer with discordant receptor status: a case report

**DOI:** 10.3389/fonc.2026.1813996

**Published:** 2026-06-26

**Authors:** Aime Ishimwe Mugisha, Gilbert Denis Umuhizi, Gerald Olwit

**Affiliations:** 1 College of Medicine and Health Sciences, School of Medicine and Pharmacy, University of Rwanda, Kigali, Rwanda; 2 Department of Clinical Oncology, Butaro Level 2 Teaching Hospital, Kigali, Rwanda; 3 Internal Medicine, Kabale University School of Medicine, Kabale, Uganda

**Keywords:** bilateral breast cancer, discordant receptor status, metachronous, neoadjuvant chemotherapy, triple-negative breast cancer

## Abstract

**Background:**

Bilateral breast cancer is uncommon, accounting for 2%–5% of cases, and presents additional clinical challenges when tumors exhibit discordant molecular subtypes. Metachronous tumors, occurring sequentially, require individualized therapeutic strategies, especially when one tumor is triple-negative and the other is hormone-responsive.

**Case presentation:**

We report on a 37-year-old woman who initially presented with a left-sided breast lump during her fifth pregnancy. Histopathology revealed invasive carcinoma of no special type (NST), progesterone receptor (PR)-positive (90%), estrogen receptor (ER)-negative, human epidermal growth factor receptor 2 (HER2)-negative, with a high proliferative index (Ki-67 = 90%), staged T2N2M0 (stage IIB/IIIA). Subsequently, she developed a right-sided breast mass, confirmed as triple-negative invasive carcinoma (T4N3M0, stage IIIC). The patient was initiated on neoadjuvant chemotherapy with the adriamycin and cyclophosphamide (AC) regimen and received supportive care, including ondansetron, morphine, and bisacodyl. Her Eastern Cooperative Oncology Group (ECOG) performance status was 1, and she experienced significant weight loss (from 68 to 46 kg in 4 months). Post-chemotherapy imaging will guide surgical planning.

**Discussion:**

This case highlights the rarity and complexity of metachronous bilateral breast cancer with discordant receptor status. Therapeutic prioritization is guided by the more aggressive tumor, in this instance, the triple-negative carcinoma, while simultaneously addressing the hormone-responsive lesion. Neoadjuvant chemotherapy allows tumor downstaging, assessment of chemosensitivity, and individualized surgical planning. Multidisciplinary management is essential to optimize outcomes and address prognostic variability between tumors.

**Conclusion:**

Metachronous bilateral breast cancer with discordant molecular subtypes presents significant diagnostic and management challenges. Individualized, biology-driven treatment strategies are critical, emphasizing the importance of systemic therapy guided by the more aggressive tumor and careful post-chemotherapy surgical planning.

## Introduction

Breast cancer is the most commonly diagnosed malignancy and the leading cause of cancer-related mortality among women worldwide, accounting for an estimated 2.3 million new cases and 685,000 deaths in 2020 (1). Its incidence varies geographically, with higher rates observed in developed countries, while mortality remains disproportionately high in low- and middle-income regions due to delayed diagnosis and limited access to specialized care (2). Breast cancer is a biologically heterogeneous disease characterized by distinct molecular subtypes defined by estrogen receptor (ER), progesterone receptor (PR), human epidermal growth factor receptor 2 (HER2), and proliferative markers such as Ki-67. These biomarkers are critical for prognosis and treatment selection, guiding the use of endocrine therapy, targeted therapy, and chemotherapy (3, 4).

Bilateral breast cancer, defined as the presence of primary tumors in both breasts, is uncommon, occurring in approximately 2%–5% of breast cancer patients (5). Bilateral tumors may present synchronously or metachronously, although the exact temporal definition varies across studies. Classical definitions describe metachronous bilateral breast cancer as contralateral tumors diagnosed more than 6 months after the initial malignancy; however, some contemporary reports and institutional practices also consider sequentially diagnosed tumors with distinct pathological confirmation as metachronous-like presentations (6). Discordant receptor status between bilateral tumors poses significant clinical challenges as each tumor may respond differently to systemic therapies and carry distinct prognostic implications (4). Among these, triple-negative breast cancer (TNBC), which is characterized by the absence of ER, PR, and HER2 expression, is particularly aggressive and is associated with early metastasis, high recurrence rates, and limited targeted treatment options (7).

The management of patients with bilateral tumors of differing molecular subtypes requires coordinated multidisciplinary involvement, including medical oncology, breast surgery, radiology, pathology, nutritional support services, and supportive care teams. In this case, the pathology findings confirming the discordant receptor profiles guided biology-driven therapeutic prioritization, while radiologic staging informed treatment sequencing and surgical planning (8). The prognostic impact of nodal involvement, tumor grade, and proliferation indices such as Ki-67 further complicates therapeutic decision-making (4, 9).

We report on a case of a 37-year-old woman who initially presented with a left-sided breast tumor during her fifth pregnancy, histopathologically confirmed as ER-negative, PR-positive, HER2-negative, and high Ki-67. Subsequently, she developed a contralateral triple-negative breast tumor. This case highlights the rarity of metachronous bilateral breast cancer with discordant molecular subtypes and underscores the clinical complexity in optimizing treatment strategies, balancing aggressive chemotherapy with considerations for hormone receptor-positive disease. Compared with previously reported cases with bilateral discordant receptor status, this report further emphasizes the practical challenges of adapting guideline-informed oncology care within constrained healthcare systems while balancing tumor biology, treatment accessibility, and patient-specific considerations.

## Case presentation

**Figure 1:**
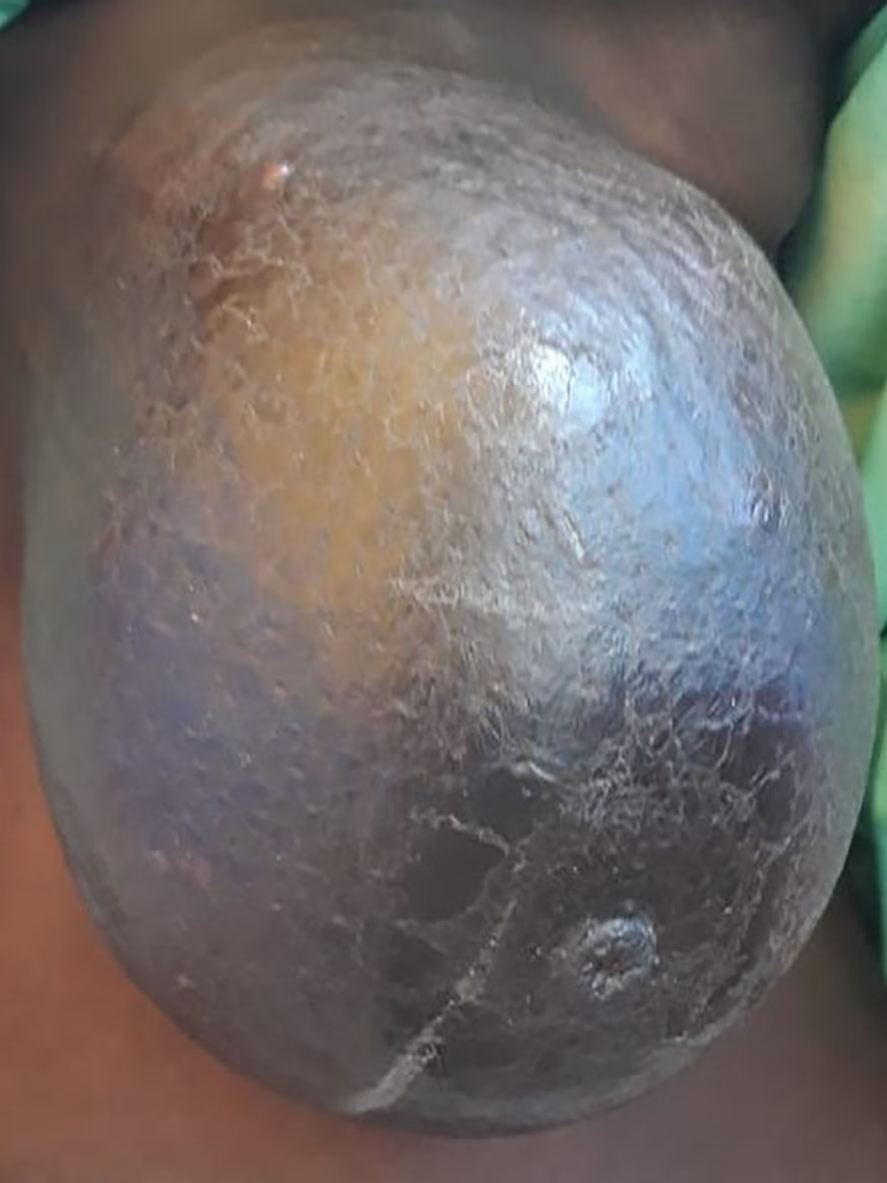
Clinical image of the left breast demonstrating significant enlargement with stretched, hyperpigmented skin and tense surface changes, consistent with locally advanced breast carcinoma.

A 37-year-old female patient initially presented with a left-sided breast lump that she first noticed during her fifth pregnancy. The mass began as a small nodule, which progressively enlarged throughout gestation. She had no prior history of breast disease and no family history of breast cancer. On clinical examination, the left breast demonstrated marked enlargement with skin stretching and hyperpigmentation, consistent with locally advanced disease, as illustrated in **Figure 1**. There was associated axillary lymphadenopathy. Core needle biopsy of the left breast confirmed invasive carcinoma of no special type (NST), Nottingham grade II, estrogenER-negative, PR-positive (90%), HER2-negative, with a high proliferative index (Ki-67 = 90%), staged as T2N2M0 (stage IIB/IIIA).

**Figure 2:**
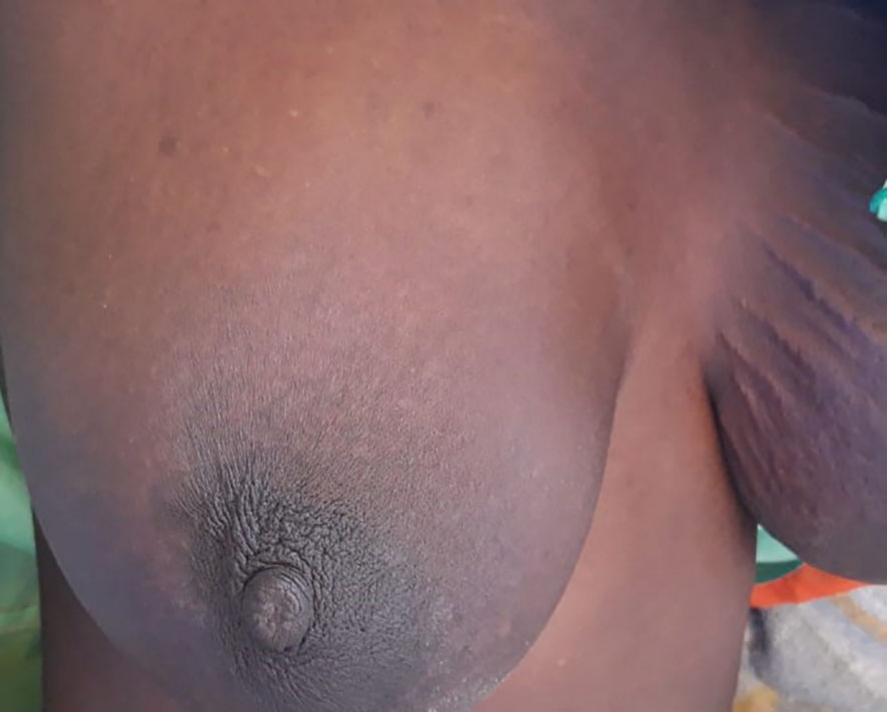
Clinical image of the right breast without obvious enlargement. Despite the absence of gross deformity, a firm palpable mass was identified on clinical examination and subsequently confirmed as invasive carcinoma.

Following histopathological confirmation of the left-sided malignancy, and during ongoing diagnostic evaluation, the patient subsequently developed a contralateral right breast mass with distinct pathological characteristics, raising consideration of a sequential bilateral primary presentation. In contrast, the right breast appeared less distorted on inspection; however, a firm palpable mass with associated axillary lymphadenopathy was identified, as shown in **Figure 2**. Biopsy of the right breast revealed invasive carcinoma with a triple-negative receptor profile (ER-, PR-, and HER2-), staged as T4N3M0 (stage IIIC), indicating a more advanced and biologically aggressive disease. Prior to the initiation of systemic therapy, a staging CT scan of the chest, abdomen, and pelvis was performed, confirming that the tumor was confined to the breast with regional lymph node involvement and no evidence of distant metastasis. Initiation of systemic therapy was deferred until pathological confirmation of the contralateral lesion was obtained.

The patient was initiated on neoadjuvant adriamycin and cyclophosphamide (AC) chemotherapy regimen, as this is the most immediately accessible and institutionally available regimen for systemic disease control. Although contemporary guideline-supported approaches for stage IIIC TNBC commonly include sequential anthracycline–taxane therapy, platinum-containing regimens, and consideration of immunotherapy, treatment selection in this case was influenced by resource availability, institutional practice patterns, drug accessibility, and the patient’s clinical condition at presentation. She is receiving supportive medications, including ondansetron, morphine, and bisacodyl. She maintains an Eastern Cooperative Oncology Group (ECOG) performance status of 1, but has experienced significant weight loss from 68 to 46 kg (height = 172 cm, body surface area = 1.48 m^2^). The markedly elevated Ki-67 index on the left-sided tumor suggests high proliferative activity, warranting close monitoring of response to therapy. Post-chemotherapy imaging reassessment is planned to evaluate tumor response and guide definitive surgical management, most likely mastectomy. At the time of reporting, the patient is currently on cycle 2 of four cycles of neoadjuvant chemotherapy with the AC regimen. She reports significant improvement in breast pain, with progressive symptomatic relief since the initiation of treatment. On interim clinical assessment, there is a noticeable reduction in tumor size, suggesting a favorable early response to chemotherapy. She continues to tolerate treatment with supportive care and remains under close multidisciplinary follow-up for ongoing evaluation of therapeutic response and planning of definitive surgical management. At the time of reporting, the patient had not yet undergone definitive breast surgery, sentinel lymph node biopsy, or axillary lymph node dissection because she remained on neoadjuvant chemotherapy. Surgical planning currently involves bilateral mastectomy with regional nodal management following completion of therapy and post-chemotherapy reassessment. Surgical pathology, including margin status and degree of tumor regression, will be evaluated following surgery. The timeline of clinical events is shown in **Table 1**.

**Table 1:** Chronological timeline of clinical presentation, diagnostic evaluation, staging, and treatment of the patient.

Date	Clinical event
August 2025	Patient noticed a small lump in the left breast during her fifth pregnancy, which progressively increased in size.
October 2025 (postpartum)	Clinical evaluation performed. Breast ultrasound and mammography revealed suspicious lesions in the left breast with associated axillary lymphadenopathy.
October 2025	Core needle biopsy of the left breast performed, confirming invasive carcinoma (NST), ER-negative, PR-positive, HER2-negative, with high Ki-67.
December 2025	Patient developed a palpable mass in the right breast with axillary lymphadenopathy. Breast imaging (ultrasound ± mammography) showed suspicious lesion.
December 2025	Core needle biopsy of the right breast performed, confirming triple-negative invasive carcinoma.
January 2026	Staging CT scan (chest, abdomen, and pelvis) performed, demonstrating disease confined to the breasts with regional lymph node involvement and no evidence of distant metastasis.
January 2026	Final diagnosis of metachronous bilateral breast cancer with discordant receptor status established.
January 2026	Initiation of neoadjuvant chemotherapy (AC regimen: adriamycin and cyclophosphamide), cycle 1
February 2026 (current)	Patient on cycle 2 of 4 of AC chemotherapy, with clinical improvement in symptoms and reduction in tumor size. Ongoing monitoring and planning for surgical management

### Timeline

## Patient perspective

The patient reported emotional distress following the diagnosis of bilateral breast cancer, particularly due to its progression during and after her fifth pregnancy. She expressed anxiety regarding the aggressive right-sided tumor and the noticeable changes in her body, including the significant weight loss.

Despite these challenges, she remains determined to continue treatment and hopeful that chemotherapy will reduce the tumor burden and allow for definitive surgical management.

## Discussion

Metachronous bilateral breast cancer is an uncommon clinical entity, representing approximately 2%–5% of breast cancer cases, and presents unique diagnostic and therapeutic challenges (1, 2). In this case, the patient initially presented with a left-sided breast tumor during her fifth pregnancy, later developing a contralateral triple-negative breast carcinoma. Bilateral tumors with discordant molecular subtypes are rare but clinically significant, as each tumor may exhibit distinct biological behavior, response to systemic therapy, and prognostic implications (10, 11). Formulating the treatment strategy presented several challenges, including the discordant receptor biology, advanced bilateral disease burden, the significant cancer-related weight loss, limited access to newer systemic therapies, and the need to balance evidence-based recommendations with locally available resources. Although guideline-supported management of stage IIIC TNBC may include anthracycline–taxane combinations, platinum-based intensification, and immunotherapy, treatment selection in this case was influenced by resource availability, financial constraints, and the patient’s clinical condition. Published reports suggest that systemic therapy in bilateral breast cancer with discordant receptor status should generally target the biologically more aggressive tumor, while additional therapies are tailored to the receptor profile of the contralateral lesion. Triple-negative tumors are commonly prioritized because of their aggressive behavior and limited targeted treatment options.

According to contemporary National Comprehensive Cancer Network (NCCN)-informed management strategies, patients with stage IIIC TNBC are frequently treated with sequential anthracycline–taxane chemotherapy, with consideration of platinum-based intensification and immunotherapy, where clinically feasible and available. In the present case, neoadjuvant AC chemotherapy was selected as the most feasible initial regimen within the limitations of local resource availability and institutional treatment pathways. Although contemporary guideline-supported approaches for stage IIIC TNBC commonly include sequential anthracycline–taxane therapy, platinum-containing regimens, and consideration of immunotherapy, treatment selection in this case was influenced by resource availability, institutional practice patterns, drug accessibility, and the patient’s clinical condition at presentation. Treatment decisions were guided by the more aggressive right breast tumor while concurrently addressing the hormone receptor-positive left breast tumor. Alternative chemotherapy regimens were considered; however, the AC regimen was chosen to provide optimal systemic control, facilitate tumor downstaging, and guide subsequent surgical management.

The left breast tumor, although hormone receptor-positive (PR = 90%) and HER2-negative, demonstrated high proliferative activity (Ki-67 = 90%), suggesting increased aggressiveness. The contralateral tumor was triple-negative, a subtype associated with higher recurrence rates, early metastasis, and limited targeted treatment options (7, 8). Discordant receptor status in bilateral disease necessitates individualized management, prioritizing systemic therapy according to the more aggressive tumor while concurrently addressing the hormone-sensitive component (10).

Neoadjuvant chemotherapy (AC regimen: adriamycin and cyclophosphamide) was selected to simultaneously target both tumors, reduce the tumor burden, assess chemosensitivity, and facilitate surgical planning (12, 13). Supportive care, including antiemetics, analgesics, and bowel management, is essential to maintain performance status and ensure treatment adherence. The sequence of therapy reflects current evidence-based strategies for bilateral tumors with divergent receptor profiles, emphasizing aggressiveness-driven systemic treatment (8, 10).

In cases of bilateral breast cancer with discordant molecular subtypes, systemic therapy is typically guided by the more aggressive tumor to optimize disease control. In this patient, the triple-negative right breast tumor dictated the initiation of neoadjuvant chemotherapy due to its higher risk of rapid progression. The hormone receptor-positive left breast tumor will be managed with adjuvant endocrine therapy following systemic chemotherapy and surgery, ensuring a comprehensive, biology-driven treatment strategy.

The patient’s severe cancer-related weight loss prompted multidisciplinary nutritional assessment and supportive interventions aimed at maintaining functional status and treatment tolerance. Symptom-directed supportive care, including analgesia, antiemetics, and bowel management, was integrated throughout therapy. The management of cancer-related cachexia should include nutritional support, symptom control, and multidisciplinary interventions as it plays a critical role in maintaining functional status, treatment tolerance, and overall quality of life. A review of the literature reveals that metachronous bilateral breast cancer with discordant receptor status is rare, with reported incidence ranging from 2% to 5% of breast cancer cases. Similar to previously reported cases, our patient exhibited contralateral triple-negative and hormone receptor-positive tumors, highlighting the challenges in systemic therapy prioritization. Comparison with prior reports underscores the importance of biology-driven treatment, individualized chemotherapy regimens, and consideration of adjuvant endocrine therapy, aligning with current evidence on optimal management strategies for such complex cases. Definitive surgical planning, most likely mastectomy with regional nodal management, was intentionally deferred pending reassessment of the treatment response following completion of neoadjuvant chemotherapy. Post-treatment imaging will additionally guide radiotherapy planning for both breasts and regional nodal basins. The absence of neoadjuvant immunotherapy and platinum-based intensification in this patient does not necessarily reflect ideal guideline-based care, but rather highlights the real-world oncology management challenges encountered in resource-limited settings, including restricted access to advanced therapies, financial limitations, and institutional treatment constraints. Because the patient remains in the neoadjuvant treatment phase, definitive postoperative histopathology, radiation therapy details, and final adjuvant treatment strategies are not yet available. Planned post-surgical management is expected to include reassessment for locoregional radiotherapy and endocrine therapy for the hormone receptor-positive tumor component. Surgical pathology following bilateral mastectomy will additionally evaluate the margin status and tumor response to neoadjuvant chemotherapy. Treatment planning required multidisciplinary coordination involving oncology, breast surgery, pathology, radiology, nutritional support, and supportive care services. Therapeutic prioritization was guided by the biologically more aggressive triple-negative tumor while also considering the hormone-responsive contralateral lesion and the patient’s overall clinical condition.

We acknowledge that the interval between tumor diagnoses in this patient was shorter than the classical >6-month definition frequently used for metachronous bilateral breast cancer. However, given the sequential clinical presentation, separate pathological confirmation, and discordant receptor biology, this presentation shares characteristics described in some contemporary reports as metachronous-like bilateral disease. We acknowledge that reporting this case during an interim treatment phase limits assessment of the radiologic response, pathologic complete response, long-term recurrence risk, and survival outcomes. Accordingly, the principal educational value of this report lies in illustrating the initial multidisciplinary therapeutic decision-making and biology-driven management challenges. An additional limitation of this report is the unavailability of the histopathology slide images and mammographic/breast MRI images during manuscript preparation, as the authors had access only to formal pathology and radiology reports. Nevertheless, all diagnoses were established from documented histopathological and radiologic findings in the patient’s medical records.

## Conclusion

Metachronous bilateral breast cancer with discordant molecular subtypes is a rare and clinically challenging presentation. The coexistence of a hormone-responsive tumor and a triple-negative tumor necessitates individualized, biology-driven management, with systemic therapy prioritized based on the more aggressive tumor. Although the patient remains in the early phase of neoadjuvant treatment without definitive surgical or long-term oncologic outcome data available at the time of reporting, this case highlights the diagnostic and therapeutic complexities associated with bilateral breast cancer exhibiting discordant receptor biology. This case emphasizes the importance of multidisciplinary care, careful monitoring, and tailored therapeutic strategies to improve outcomes in patients with complex bilateral breast malignancies. Clinicians should be aware of the potential for discordant tumor biology in bilateral breast cancer as it directly influences the prognosis, treatment decisions, and patient counseling. The present report highlights not only the biological heterogeneity of bilateral breast cancer but also the complexity of delivering individualized oncology care in resource-constrained settings, particularly in young patients presenting during the pregnancy/postpartum period.

## Data Availability

No dataset available, as this is a case report. Requests to access the datasets should be directed to Aime Ishimwe Mugisha, mugishaaime456@gmail.com.
